# Voluntary nutrition guidelines to support healthy eating in recreation and sports settings are ineffective: findings from a prospective study

**DOI:** 10.3934/publichealth.2018.4.411

**Published:** 2018-11-08

**Authors:** Jessie-Lee D McIsaac, Sherry Jarvis, Dana Lee Olstad, PJ Naylor, Laurene Rehman, Sara FL Kirk

**Affiliations:** 1 Healthy Populations Institute, Dalhousie University, PO Box 15000, 1318 Robie Street, Halifax, NS B3H 4R2 Canada; 2 Faculty of Education and Department of Child and Youth Study, Mount Saint Vincent University, 166 Bedford Highway, Halifax, Nova Scotia, B3M 2J6; 3 Department of Community Health Sciences, Cumming School of Medicine, University of Calgary, 3280 Hospital Drive NW, Calgary, AB T2N 4Z6 Canada; 4 School of Exercise Science, Physical and Health Education, University of Victoria, PO Box 3015 Stn CSC, Victoria, BC V8W 3P1 Canada; 5 School of Health and Human Performance, Dalhousie University, Stairs House, PO Box 15000, 6230 South Street, Halifax, NS B3H 4R2 Canada

**Keywords:** children, food environments, recreation and sports settings, nutrition, guideline implementation, public policy

## Abstract

Interventions to support healthy eating among populations are needed to address diet-related chronic disease. Recreation and sport settings are increasingly identified as ideal settings for promoting overall health, particularly for children, through creation of environments that support positive health behaviours. These publicly funded settings typically support health through physical activity promotion. However, the food environment within them is often not reflective of nutrition guidelines. As more jurisdictions release nutrition guidelines in such settings, the purpose of this study was to assess whether voluntary nutrition guidelines, released in 2015 in the Canadian province of Nova Scotia, had any impact on food environments in these settings. Baseline and follow-up audits of food environments were conducted one year before (in 30 facilities) and one year after guideline release (in 27 facilities). Audits involved classifying all foods and beverages within vending machines and concessions as *Do Not Sell*, *Minimum*, *Moderate*, or *Maximum* nutrition, using criteria provided in the guidelines. The proportion of items within each category was calculated, and differences from pre- to post-guideline release were assessed using Chi-squared statistics. Results indicated limited change in food and beverage provision from pre- to post- guideline release. In fact, from pre- to post-guideline release, the proportion of *Do Not Sell* vending beverages and concession foods increased significantly, while *Maximum* concession beverages decreased, suggesting a worsening of the food environment post-guideline release. Findings suggest that voluntary guidelines alone are insufficient to improve food environments in recreation and sport settings. For widespread changes in the food environment of these settings to occur, more attention needs to be paid to reducing social, cultural, political and economic barriers to change (real and perceived) that have been identified in these settings, alongside developing leadership and capacity within facilities, to ensure that positive changes to food environments can be implemented and sustained.

## 1. Introduction

Interventions to support healthy eating among populations are needed to address diet-related chronic disease [1],[2]. Because environmental and social determinants influence individual eating behaviours, the implementation of policies or guidelines to support healthier food environments offer potential to improve population-level health outcomes [2],[3]. Alongside schools, recreation and sport settings (RSS) are increasingly identified as ideal settings for promoting overall health among children, through creation of environments that support positive health behaviours [4]–[8]. These publicly funded settings offer context for engaging a variety of community members with health messaging, including recreation participants spanning all ages and abilities [4]. Yet, while these settings may promote health in the form of physical activity, they are often characterized by unhealthy food environments with high availability of energy-dense, nutrient-poor, processed foods that are quick to prepare and inexpensive to provide, and limited availability of nutrient-rich, whole, minimally processed foods [9]–[14].

In Canada, RSS are typically funded at the community level, whereas supporting changes within the broader food environment often rests at the provincial level. Some provinces have released nutrition guidelines for RSS to support provision of healthy foods within these settings [13],[15]. Such an approach was recently undertaken in the Canadian province of Nova Scotia with the release of the Healthy Eating in Recreation and Sport Settings (HERSS) voluntary guidelines in the Fall of 2015 [16]. These guidelines were developed in partnership with stakeholders from health and recreation organizations across Nova Scotia and align with the province's existing school food and nutrition policy [16]. The primary objectives of the HERSS guidelines are to create and support an environment where people have increased access to healthy foods and beverages. Two other Canadian provinces previously released voluntary nutrition guidelines for RSS. In 2008, the Alberta Nutrition Guidelines for Children and Youth were released [11],[12], while in British Columbia the Healthier Choices in Vending Machines in BC Public Buildings Policy came into effect in 2006 and was updated in 2014 to align with other provincial nutrition standards [9],[10]. Although a number of studies have examined the role of these guidelines in supporting healthier food environments [9]–[12], none were able to assess food environment quality prior to guideline release, which limits understanding of their potential impact.

The purpose of this study was to assess whether voluntary nutrition guidelines, released in 2015 in the Canadian province of Nova Scotia, had any impact on food environments in RSS. This study is the first, to our knowledge, to prospectively examine any change in the quality of the food environment in RSS from pre- to post-guideline release. This information will contribute towards a more robust understanding of the potential impact of voluntary nutrition guidelines for RSS in other jurisdictions.

## 2. Materials and Methods

### 2.1. Study design

We employed a prospective study design to assess the food environment in a sample of RSS from across Nova Scotia one year prior to (Fall 2014) and one year following (Fall 2016) the release of the voluntary HERSS guidelines (Fall 2015). Ethical approval to conduct the audits was provided by the Dalhousie University Research Ethics Board.

Food and beverage environment audits were conducted in facilities that were selected to represent the six sport and recreation regions within the province at the time of baseline data collection (2014). Data were collected and analyzed from 30 different RSS out of a total of 106 RSS across the province (28.3% of provincial RSS). These RSS were selected in consultation with RSS representatives from each sport and recreation region. Representatives were asked to validate a list of RSS within their region and identify RSS to participate in the audits based on number and types of users. If a regional representative did not list enough RSS to fulfill the quota determined by the sampling frame, the research team purposively selected additional facilities to ensure broad representation of RSS. Regional representatives rarely identified indoor aquatic facilities as good candidates for participation, and therefore these were only sampled in the event that regional representatives did not identify enough facilities to fulfill the sampling frame. A variety of RSS were audited, including arenas (e.g., ice rinks) and multi-use facilities (e.g., facilities offering a variety of services such as ice rinks, indoor pools, gymnasiums, fitness centres, walking tracks, etc.). Follow-up audits were completed at 27 of the 30 RSS audited at baseline. Of the three that did not participate in the follow-up audits, one was no longer operational, while two did not respond to follow-up audit requests.

### 2.2. Data collection

Audits were conducted of all vending machines and concessions in each facility by a research assistant (some facilities did not have a concession). The location of each vending machine was recorded to ensure that the same machines were audited across each phase of the research. Details of all foods and beverages available in vending machines and concessions were recorded, including product name and manufacturer, product description, size, method of preparation/cooking and price. In vending machines, where multiple slots were filled with the same brand or type of product, it was counted each time it was displayed to provide a measure of how much space was devoted to each type of food or beverage item. Photographs of each vending machine were also taken to verify the products recorded during the environmental audit for future reference. Concession products were recorded by reviewing the menus at each facility and by observation if the concession was operating during the audit. Photographs were also taken of the menus for future reference.

### 2.3. Analysis

**Figure 1.**
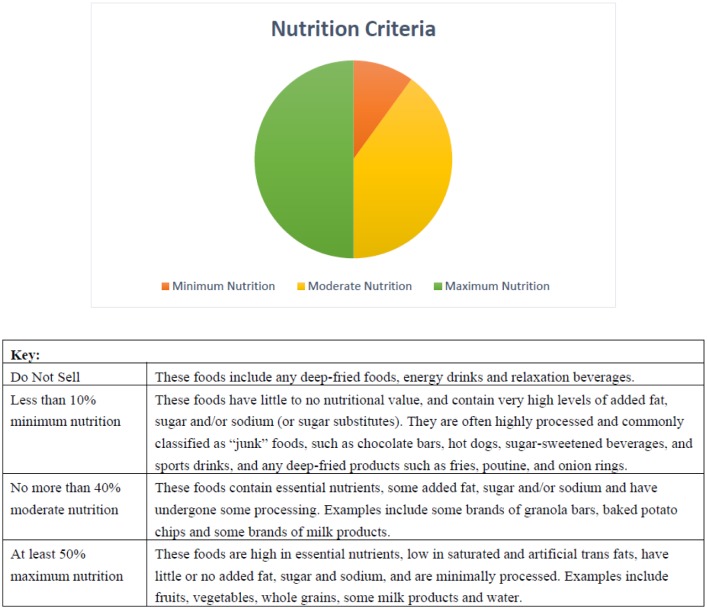
Healthy Eating in Recreation and Sport Settings Nutrition Criteria

Food and beverage products were classified as *Do Not Sell*, *Minimum*, *Moderate*, or *Maximum* nutrition using the HERSS guideline categories, which were in turn based on the Food and Nutrition policy for NS Public Schools [17] (Figure 1). Classification of items that were not listed in the policy documents was accomplished by obtaining nutrition information from the Canadian Nutrient File [18]. The proportion of items within each category was calculated. Differences in the proportion of items within each category from pre- to post-guideline release were assessed using Chi-squared statistics. SPSS (version 24; IBM Corporation, Armonk, NY) was used for all analyses, with *p* < 0.05 indicating significant differences.

## 3. Results

At baseline, audits were conducted on 30 facilities, including 916 vending food products from 28 vending machines, 2147 vending beverage products from 86 vending machines, 469 concession food products, and 175 concession beverage products. At follow-up, audits were conducted on 27 facilities, including 695 vending food products from 23 vending machines, 1869 vending beverage products from 68 vending machines, 514 concession food products, and 153 concession beverage products.

Overall, at both baseline and follow-up, the majority of vending foods and beverages audited were of *Minimum* nutrition, while concession products consisted primarily of *Minimum* and *Do Not Sell* items (Table 1). Furthermore, it can be seen that the food environment of the facilities audited actually worsened post-guideline. While there was no significant change in vending foods, the proportion of *Do Not Sell* and *Moderate* nutrition concession foods actually increased significantly post-guideline release. For beverages, the proportion of *Do Not Sell* vending beverages and *Moderate* concession beverages were significantly higher post guideline release, while the proportion of *Maximum* nutrition concession beverages was significantly lower, all being in the opposite direction of what would be expected.

**Table 1.** Mean proportionate availability of foods and beverages in RSS before and after the release of guidelines.

Product Type	Category	Pre-guideline % (n=30)	Post-guideline % (n=27)	Chi-Square Test	
				*X^2^*	*P value*
Vending Foods	Do Not Sell	0	0	-	-
	Minimum	91	86	114.3	NS^1^
	Moderate	9	14	101.5	NS
	Maximum	0.1	0.1	-	-
Vending Beverages	Do Not Sell	2	3.6	80.0	*P* < 0.001
	Minimum	72	71	460.0	NS
	Moderate	0.2	0.6	0.52	NS
	Maximum	26	25	419.7	NS
Concession Foods	Do Not Sell	26	32	220.0	*P* < 0.001
	Minimum	50	42	304.0	NS
	Moderate	16	18	200.0	*P* < 0.001
	Maximum	8	8	-	-
Concession Beverages	Do Not Sell	0	0	-	-
	Minimum	65	67	126.3	NS
	Moderate	10	11	110.6	*P* < 0.001
	Maximum	25	22	111.1	*P* < 0.05

Note: NS = non-significant.

## 4. Discussion

With an increased focus on the need for healthier food environments in settings where children spend their time [1],[4],[11],[14], and the release of nutrition guidelines for RSS in other Canadian provinces, including British Columbia and Alberta, this study sought to describe the food environment in Nova Scotia's RSS before and after the release of voluntary nutrition guidelines. Our findings revealed that the Nova Scotia voluntary guidelines were not associated with positive changes in food and beverage provision. In fact, from pre- to post-guideline release, the proportion of *Do Not Sell* vending beverages and concession foods actually went up, while *Maximum* concession beverages went down, suggesting a worsening of the food environment over the duration of the study.

The reasons for these findings are likely complex and require some consideration of the context within the settings under study at the time of guideline release. First, it is important to consider the political context in which this study was situated. The provincial HERSS guidelines were developed in response to a provincial childhood obesity prevention strategy, called *Thrive! A Plan for a Healthier Nova Scotia*, released in 2012 [19]. This initiative aimed to help create supportive environments where people play, learn, work, and grow [19]. One of four strategic directions of *Thrive!* was to create more opportunities to eat well and be active, including supporting public policies in RSS. Through dedicated funding for *Thrive!*, the provincial government offered funding to some RSS across the province to support the introduction of healthy menu items in concessions. During baseline environmental audits for the current study, several arenas were already offering a modified menu which appeared to have had the desired effect of facilitating positive change within these facilities. However, the short-term nature of this funding, alongside a shift in political leadership and priorities that occurred through government restructuring in 2015–2016, may have led to a reduced focus on healthy eating policy, thus contributing to the worsening of the food environment identified in this study. It is already well-established that capacity-building and leadership are critical community-level implementation factors [20] but both appeared to be lacking in this provincial context. This highlights the importance of provincial, as well as municipal and facility-level, leadership. Indeed, the need for a non-partisan approach to policy aimed at supporting behavior change has previously been noted in order to ensure healthy public policies can be maintained across shifting political landscapes [21].

It is possible that there was a lack of capacity and accountability within RSS to ensure that appropriate changes to food and beverage provision were made and maintained. Related to political context, it can be seen that provincial funding available over the time of baseline data collection may have improved the capacity of some RSS to implement changes in the food environment prior to the release of the guidelines, but when this funding ended, any such improvements could not be maintained and are therefore reflected in the worsening of the food environment that we observed. Furthermore, our qualitative analysis prior to the release of the guidelines revealed that, while RSS staff valued healthy eating and wanted to provide healthier options for patrons, they expressed financial concerns (e.g. fear of losing revenue if unhealthy foods and beverages are no longer available), and a perception that dietary behaviours were a matter of personal choice, which may have impacted their willingness to implement the guidelines on their release [22]. In addition, the cultural norms associated with RSS (e.g., a desire for unhealthy foods at arenas), remained a significant contributing factor to the unhealthy food environments in RSS, through a real or perceived influence on patron demands for certain types of foods and beverages and expectations of what should be available at special events (e.g., hockey games or celebrations) [22]. It is worth noting, however, that the assumption that patrons are unwilling to choose healthy food options in these settings is not supported when patron perspectives are considered alongside those of RSS staff [23].

Related to capacity and accountability, it is possible that facility personnel tasked with interpreting the guidelines made changes that were perceived to be beneficial, but which did not have the desired impact as it relates to the guideline categories. For example, several facilities replaced soft drinks with 100% juice products, however because these were offered in >250ml bottles they were still classified as *Minimum* nutrition. Similarly, foods like baked chips or granola bars could be classified as *Moderate* or *Minimum*, depending on their ingredients. Some facilities reduced their food and beverage vending machines, as can be seen by the lower number of these available in the follow-up audits. However, while guideline-compliant beverages like water and milk were available in many RSS, *Minimum* nutrition beverages remained ubiquitous before and after guideline release. It is also noteworthy that the proportion of *Do Not Sell* vending beverages (e.g. energy drinks) increased slightly at several of the facilities on university campuses, possibly reflecting patron demand for these newer types of drinks, even though they are not compatible with the guidelines. In addition, one facility installed a deep-fat fryer between baseline and follow-up data collection. These changes may explain why we saw shifts in the proportions of foods and beverages across the *Minimum*, *Moderate* and *Do Not Sell* categories, but not reflected in the *Maximum* nutrition categories. It is also encouraging that there were no *Do Not Sell* vending foods or concession beverages pre- and post-guideline release, although the reason for this is unclear, but, again, may reflect changes implemented at the time of baseline data collection, through the *Thrive!* funding previously noted.

The worsening of the food and beverage environment in RSS across Nova Scotia included in this study means that unhealthy food and beverages remain readily available to patrons of these facilities. Although increasing healthy food options, which is a goal of the HERSS voluntary guidelines, may improve the overall availability of healthy items, the continued overwhelming presence of unhealthy options may limit their impact. This is particularly concerning for children and youth, because studies have shown that when healthy and unhealthy choices are available within RSS, children and youth continue to purchase primarily unhealthy options [6],[24]. We did not specifically assess food marketing within the RSS studied, but it is already known that many unhealthy foods are targeted specifically to children and youth as ‘kid food’ [25] and eating junk food has been associated with independence from adults, friendship, and youth [26]. The availability of unhealthy foods in RSS may be so entrenched in our culture that they are normalized. Thus, a multifaceted approach is critical to address the difficult task of denormalizing unhealthy foods and promoting healthier items in RSS [27]. Our findings suggest that voluntary nutrition guidelines alone are insufficient to catalyze the degree of change necessary to support health. Instead, structural changes are needed, including mandatory policies that are implemented, monitored and enforced to ensure that the healthiest choices are easier to make [28]. This is particularly important to consider, given that the facilities we surveyed were publicly funded and the costs associated with undermining health in these settings may ultimately be borne by the publicly funded health care system through increased costs associated with managing chronic disease.

## 5. Strengths and limitations

A strength of our study is the prospective design that allowed baseline measures of the food environment to be conducted one year prior, and one year after the release of voluntary province-wide guidelines. Further, the sampling of RSS across different regions of the province, as well as a variety of facility types (e.g., multipurpose, arenas, etc.) allowed us to capture the diversity of food environments within the provincial RSS context, particularly given that more than a quarter of all RSS in the entire province were assessed. This represents a real-world implementation of voluntary guidelines, thereby providing important learnings for guideline implementation elsewhere in Canada.

A key limitation is that there was no comparison group. Because the guidelines were intended for province-wide release, a comparison group from within the province was not feasible. Therefore, we cannot account for secular change or attribute any changes to the nutrition guidelines, nor can we disentangle the impact of *Thrive!* funding that was available to some RSS prior to baseline. Another important limitation is that we did not assess specific facility-level barriers and enablers to implementation, although previous research that has done so has identified similar challenges to those noted here [10],[12].

## 6. Conclusion

The results of this study highlight that voluntary nutrition guidelines had no positive impacts on RSS food environments in Nova Scotia. This suggests that voluntary nutrition guidelines, without additional support, such as capacity building or accountability measures, may be ineffective. For widespread changes in the food environment of RSS to occur, more attention needs to be paid to reducing social, cultural, and economic barriers to change (real and perceived) that have been identified in these settings, alongside developing leadership and capacity within facilities, to ensure that changes can be implemented and sustained.
